# Genome-Wide Association Reveals Trait Loci for Seed Glucosinolate Accumulation in Indian Mustard (*Brassica juncea* L.)

**DOI:** 10.3390/plants11030364

**Published:** 2022-01-28

**Authors:** Erwin Tandayu, Priyakshee Borpatragohain, Ramil Mauleon, Tobias Kretzschmar

**Affiliations:** Faculty of Science and Engineering, Lismore Campus, Southern Cross University, East Lismore, NSW 2480, Australia; e.tandayu.10@student.scu.edu.au (E.T.); priyakshee.borpatra.gohain@scu.edu.au (P.B.); ramil.mauleon@scu.edu.au (R.M.)

**Keywords:** *Brassica juncea*, genome-wide association studies, glucosinolates (GSL), seed quality

## Abstract

Glucosinolates (GSLs) are sulphur- and nitrogen-containing secondary metabolites implicated in the fitness of Brassicaceae and appreciated for their pungency and health-conferring properties. In Indian mustard (*Brassica juncea* L.), GSL content and composition are seed-quality-determining traits affecting its economic value. Depending on the end use, i.e., condiment or oil, different GSL levels constitute breeding targets. The genetic control of GSL accumulation in Indian mustard, however, is poorly understood, and current knowledge of GSL biosynthesis and regulation is largely based on *Arabidopsis thaliana*. A genome-wide association study was carried out to dissect the genetic architecture of total GSL content and the content of two major GSLs, sinigrin and gluconapin, in a diverse panel of 158 Indian mustard lines, which broadly grouped into a South Asia cluster and outside-South-Asia cluster. Using 14,125 single-nucleotide polymorphisms (SNPs) as genotyping input, seven distinct significant associations were discovered for total GSL content, eight associations for sinigrin content and 19 for gluconapin. Close homologues of known GSL structural and regulatory genes were identified as candidate genes in proximity to peak SNPs. Our results provide a comprehensive map of the genetic control of GLS biosynthesis in Indian mustard, including priority targets for further investigation and molecular marker development.

## 1. Introduction

Glucosinolates (GSLs) are a class of well-studied sulphur (S)- and nitrogen (N)- containing secondary metabolites almost exclusively found in Brassicaceae, which include the economically and nutritionally important crops *B. napus* (canola and rapeseed), *B. juncea* (Indian mustard), *B. oleracea* (cabbage) and *B. rapa* (Chinese cabbage, turnip) [1,2,3]. Most of our knowledge on GSL biosynthesis, its regulation and its links to other metabolic pathways is based on the closely related model plant, *Arabidopsis thaliana* [1,4,5]. GSLs are categorised into three major classes, depending on the amino acid they are derived from: (i) aliphatic GSLs, predominantly derived from methionine and, to a lesser extent, from leucine, isoleucine and valine; (ii) aromatic GSLs, mostly derived from phenylalanine or tyrosine and (iii) indolic GSLs, derived from tryptophan. The synthesis of GSLs proceeds in three major steps: (i) chain elongation of precursor amino acids (only for methionine and phenylalanine), (ii) GSL core structure formation and (iii) GSL side chain modification. A recent comprehensive inventory from the literature and pathway databases (KNApSAcK, KEGG and AraCyc) listed as many as 113 genes associated with GSLs in *Arabidopsis* that were identified and characterised over the last two decades [4]. This includes 53 biosynthetic genes found in the KEGG or AraCyc databases, 32 experimentally confirmed biosynthetic genes, 23 transcriptional components and five transporters. While the GSL biosynthetic pathways are well understood in *Arabidopsis*, the respective regulatory and metabolic networks in the allotetraploid Brassica crops (*B. napus* and *B. juncea*) are suggested to be much more complex due to their intricate evolutionary history [6].

Indian mustard is an economically important *Brassica*, cultivated for two distinct markets. In India, Bangladesh, China and the Ukraine, and more recently in Canada and Australia, it is grown as an oilseed crop [7]. On the other hand, in Europe, North America, Argentina and China, it is primarily grown for condiment production (e.g., mustard and “wasabi” paste). Both end uses rely on GSL content as a trait to be selected either for or against during varietal improvement. “Canola” is a trademark term of the Canadian Canola Association used to describe rape or oilseed cultivars with “double low” GSLs (<30 µmol/g in defatted seed meal) and less than 2% erucic acid [8]. In *B. juncea* grown as a canola-type oilseed crop, GSLs have largely been selected against, which enables seed meal to be used for animal feed after oil extraction. Breeding for low-GSL *B. juncea* was spearheaded by Canadian breeders through the introgression of low-GSL “Bronowski” alleles from canola *B. napus* into an Indian high-GSL *B. juncea* line [9]. The resulting donor genotype for the low-GSL trait has been extensively used in breeding for low GSLs in Canadian and Australian germplasm [10]. As such, canola-quality *B. juncea* has become a viable alternative oil crop [11,12,13]. For the condiment market, high GSL levels, high sinigrin in particular, are desirable [14]. Sinigrin, when hydrolysed, produces allyl-isothiocyante (AITCs), including sulphoraphane, responsible for the pungency of mustard and demonstrated to possess tumour suppression properties [15,16]. Notably, Indian mustard predominantly accumulates the aliphatic GSLs 2-propenyl-GSL (sinigrin) and 3-butenyl-GSL (gluconapin), and, to a lesser extent, 2-hydroxy-3-butenyl-GSL (progoitrin) [14,17,18].

Enhancing the health-beneficial GSL levels in varieties aimed for vegetable or condiment use and reducing the overall GSL and erucic acid levels, while increasing desirable fatty acids in oilseed cultivars, remain among the key seed quality traits for *B. juncea* variety improvement [17]. A better understanding of the genetic bases of trait variation and corresponding beneficial alleles would aid in the development of molecular markers for varietal improvement and an accelerated rate of genetic gain [19,20]. Earlier, classical QTL mapping deciphered beneficial allelic variations for seed quality traits in *B. juncea* [21,22,23]. Recently, genome-wide association study (GWAS) has become the more popular choice to dissect the genetic basis of these complex traits. Compared with classical quantitative trait locus (QTL) mapping, which is generally confined to alleles and novel recombination within a bi-parental population, GWAS is able to tap into the allelic pool of broader populations that have undergone natural and artifical selection throughout domestication history. Since GWAS utilises a broader allelic pool, more variation is investigated. Furthermore, actual causal variants tend to be much closer to detected associated markers in GWAS, owing to the longer recombination history than in the case of a bi-parental population. As a result, GWAS offers a higher mapping resolution of the underlying genomic regions associated with the trait of interest. In *Brassica* crops, GWAS has been successfully employed for dissecting the genetic architecture of seed quality traits such as GSL accumulation, fatty acid composition and shattering resistance [24,25,26,27,28]. In *B. juncea*, high-density single-nucleotide polymorphisms (SNPs) were identified through different strategies, including double-digest restriction-associated DNA (dd-RAD) [29], RNA sequencing [30], specific-locus amplified fragment sequencing (SLAF-seq) [31], genotyping-by-sequencing (GBS) [29] and resequencing [17]. With these, GWAS has been utilised to investigate seed GSL content using high-density SNPs [17,32]. Akhatar et al., 2020, employed GWAS for seed quality traits including GSL content at varying nitrogen levels under field conditions, while Yang et al., 2021, performed GWAS on a set of vegetable and oilseed *B. juncea*, in conjunction with deploying two new genome sequences representing vegetable and oilseed varieties. Among the candidate genes proposed in these studies, only a *MYB28*, a major regulatory gene for aliphatic GSL biosynthesis, could be linked to the current inventory [4] of GSL genes in *Arabidopsis*. This suggests that a large number of possible genetic mechanisms may yet be uncovered through GWAS. Thus, the aim of this study was to perform GWAS on a set of oilseed Indian mustard to further elucidate the genetic basis and add to the current understanding of seed GSL accumulation in Indian mustard.

## 2. Results

### 2.1. Genotype Data

A total of 69,594 SNP sites, with 61,931 (89%) anchored onto chromosomes, was obtained from the variant calling. An initial filtering for SNPs anchored onto chromosomes for 60% call rate, non-maf (minor allele frequency) filtered and 10% maximum marker heterozygosity resulted in 15,263 SNPs (26% overall with missing SNP calls), and missing states were imputed. Following imputation, a final set of 14,125 SNPs resulted from filtering for 5% minor allele frequency and 20% maximum heterozygosity and was used for GWAS. 

### 2.2. Cluster, Population Structure and Principal Component Analyses of B. juncea Diverse Panel

**Figure 1:**
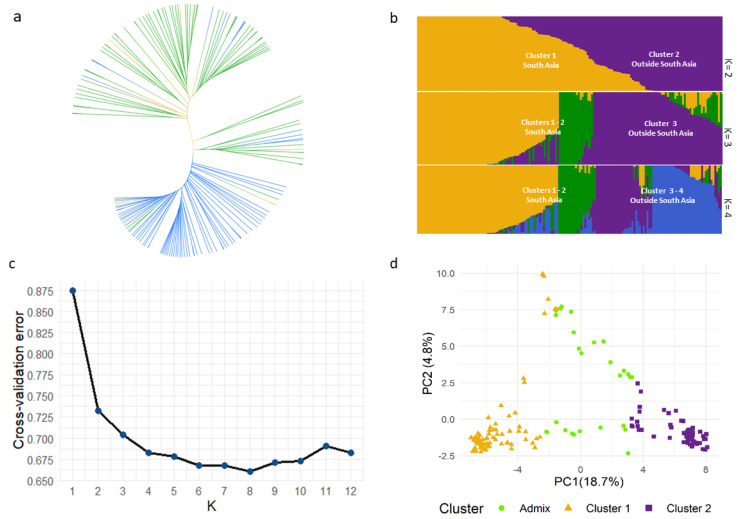
(**a**) Cluster analysis based on genetic distance using an UPGMA tree with branches coloured based on geographical origin: India and Pakistan and rest of South Asia (blue), rest of Asia, Europe, North America, Africa and Australia (green) and unknown origin (yellow). (**b**) Population structure as depicted by a sorted bar plot of ancestry proportions for K = 2–4, inferred with ADMIXTURE. (**c**) Ten-fold cross-validation error of ADMIXTURE analyses of K = 1 to 12. (**d**) Principal component analysis (PCA) coloured based on cluster assignment (threshold of 70% membership probability) at K = 2 in ADMIXTURE. Orange triangles used for cluster 1, purple squares for cluster 2 and green dots for admixture cluster.

The diversity panel consisted of 158 accessions from 28 countries, representing South Asia (53%, mostly from India and Pakistan), Asia (13%, other than South Asia), Europe (11%), North America (6%), Australia (6%), Africa (6%) and unknown origin (8%) (Table S1). Three approaches—(i) hierarchical clustering, (ii) population structure and (iii) PCA—revealed a genetic structure composed of two population clusters broadly reflecting geographical origin. UPGMA-based hierarchical clustering revealed one major cluster comprising accessions from the South Asian countries of India and Pakistan (blue-coloured branches), while the other major cluster contained accessions from outside of South Asia (green-coloured branches) (Figure 1a). Not all lines, however, matched this trend, including a few accessions from India, Nepal, Afghanistan and Bangladesh that located within the outside-South-Asia cluster and a few entries from Europe, Zimbabwe and China that fell within the South Asia cluster. A third minor cluster was largely composed of accessions from China and a few from Bhutan. ADMIXTURE suggested a similar structure as UPGMA (Figure 1b). At K = 2, cluster 1 was composed of accessions from India and Pakistan, while cluster 2 was mostly composed of accessions from outside India and Pakistan, a trend consistent with a previous report [20]. Using a 70% membership probability cut-off at K = 2, 46% of accessions fell into cluster 1 while 37% of accessions fell into cluster 2, and the remaining 17% were classified as admixed samples. The admixed samples comprised 13 South Asian (India, Bangladesh, Afghanistan, Nepal and Bhutan) accessions and 14 accessions from outside South Asia. With increasing K until K = 4, geographical origin was still traceable to clustering. At K = 3, accessions from India and Pakistan were dispersed into clusters 1 and 2, while accessions from outside India and Pakistan mostly constituted cluster 3. This was similar at K = 4, with further sub-structuring of accessions from outside India and Pakistan comprising clusters 3 and 4. A ten-fold cross validation error plot of ADMIXTURE runs using K = 1 to 12 (Figure 1c) showed that the error started to plateau at K = 4, suggesting this as a sensible K choice, while the lowest error was observed at K = 8. A PC plot reflecting the K = 2 assignment of ADMIXTURE clearly separated the two clusters at PC1 with admix samples interspersed between the clusters (Figure 1d). Further, only 18.7% of variation was explained by PC1, with succeeding PCs explaining less than 5% of variation.

### 2.3. Variance Components, Basic Descriptive Statistics and Correlations between Total GSLs, Sinigrin and Gluconapin 

**Figure 2:**
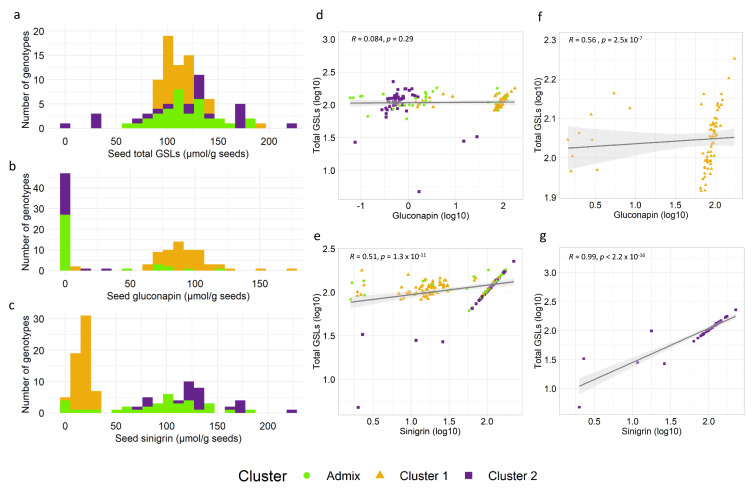
Distribution of raw mean values of (**a**) total GSLs, (**b**) gluconapin and (**c**) sinigrin, reflecting the ADMIXTURE cluster assignment at K = 2 of each accession (orange for cluster 1, purple for cluster 2 and green for admixture cluster). Correlations using log-transformed values of (**d**) gluconapin and total GSLs and (**e**) sinigrin and total GSLs in the full diversity panel. Correlation using log-transformed values of (**f**) gluconapin and total GSLs in cluster 1 and (**g**) sinigrin and total GSLs in cluster 2. Orange used triangles for cluster 1, purple squares for cluster 2 and green dots for admixture cluster.

Residual distribution showed an approximately normal distribution with a mean of zero for total GSLs, sinigrin and gluconapin (Figure S1). Sinigrin and gluconapin combined accounted for ~95–99% of the total GSLs for nearly all samples in the diverse panel (Supplemental File S1). Nearly the entire proportion of variation for total GSLs, sinigrin and gluconapin concentrations was accounted for by the samples, based on variance components analysis using Restricted Maximum Likelihood (REML) (Table S2). This was further reflected by high broad heritability values of ~98% for the single major GLSs sinigrin and gluconapin, and a slightly lower value of ~88% for the total GSLs. Sinigrin had a higher range of concentrations (1.61–225.09 µmol/g^−1^) compared to gluconapin (0.01–174.57 µmol/g^−1^). However, the gluconapin concentration was more variable, with a coefficient of variation (CV) of 108% compared to sinigrin concentrations with a CV of 79.61%. Figure 2 reflects the distribution of raw values of total GSLs (Figure 2a), gluconapin (Figure 2b) and sinigrin (Figure 2c) concentrations matched with the cluster assignment from ADMIXTURE. Notably, accessions in different clusters accumulated different single major GSLs (Figure 2b,c). As reflected in the distributions, cluster 1 and a few admixed samples predominantly accumulated gluconapin, while the majority of cluster 2 and admixed samples lacked gluconapin. Contrastingly, cluster 2 and the majority of admixed samples predominantly accumulated sinigrin, while most of cluster 1 still accumulated sinigrin at the lower ranges (Figure 2c). Given this finding, we compared the correlations of sinigrin and gluconapin with total GSLs in the full panel and within the clusters in which it predominantly accumulated (Figure 2d–g). Gluconapin had a weak correlation (r = 0.08, non-significant) with total GSLs in the full panel (Figure 2d) and a moderately positive correlation (r = 0.56) in cluster 1 (Figure 2f). A few outlier points in cluster 1 (Figure 2f) accumulated high sinigrin as their major GSL. While there was only a moderate correlation (r = 0.51) between sinigrin and total GSLs in the full panel (Figure 2e), a near perfect positive correlation (r = 0.99) was observed in cluster 2 (Figure 2g). The five outlier samples in cluster 2 (Figure 2g) comprised four accessions accumulating lower sinigrin concentrations, although it was still their major GSL, and one accession that accumulated high gluconapin as its major GSL. There were non-significant weak correlations of sinigrin and gluconapin with total GSLs within the clusters where they were not predominantly accumulated (Figure S2a,b). Sinigrin and gluconapin had significant negative correlations, having the strongest negative value (r = −0.64) in the full panel and a weak (r = −0.37) to moderate (r = −0.37) value within clusters 1 and 2, respectively (Figure S2c–e).

### 2.4. GWAS Using Multiple Models

**Figure 3:**
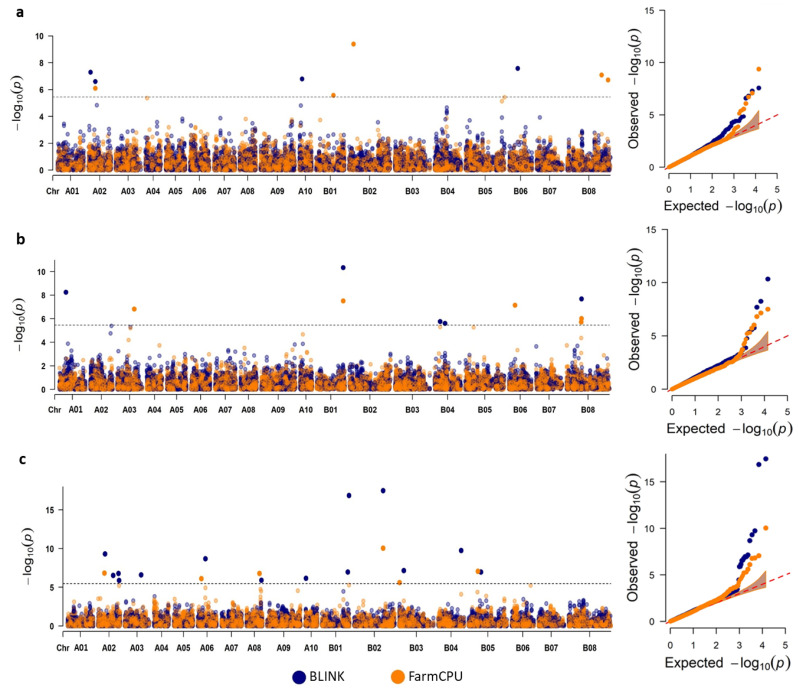
Manhattan and q-q plots for the GWAS of (**a**) total GSLs, (**b**) sinigrin and (**c**) gluconapin using BLINK (purple dots) and FarmCPU (orange dots) models. The horizontal line represents significance threshold at 5% after Bonferroni multiple test correction (−log10 (*p*) = 5.45).

Four GWAS models were tested and resulting q-q plots in each traits (Figure S3) were compared to assess which models best limited spurious associations, due to structure and relatedness. BLINK and FarmCPU returned a better correlation between observed and expected −log10 *p*-values in the lower range and returned a limited number of deviations at high log10 *p*-values. SUPER returned highly inflated −log10 *p*-values even in the lower ranges, suggestive that many detected loci were from spurious associations, which might also explain the exceptionally high number of significant SNPs detected under this model (Supplemental File S2). MLMM, despite detecting the lowest number of associations, also showed *p*-value inflation to some degree. The Manhattan plots from BLINK and FarmCPU (Figure 3) displayed a number of single SNPs associated above the Bonferroni threshold for total GSLs (Figure 3a), sinigrin (Figure 3b) and gluconapin (Figure 3c). BLINK detected four associated SNPs with the total GSL concentration, two on A02 and one each on A10 and B06, while FarmCPU detected four, one each on chromosomes A02, B01 and B02 and two on B08 (Figure 3a). One association at SNP A02_11235033 was detected in all four models (−log10 (*p*) = 6.09–10.39). The association at B02_725738 from FarmCPU was the strongest association (−log10 (*p*) = 9.38) for total GSLs considering only the BLINK and FarmCPU models. For sinigrin concentration, five SNPs were associated in BLINK, one SNP each on chromosomes A01, B01 and B08 and two on B04 (Figure 3b). FarmCPU also detected five associated SNPs, one each on chromosomes A03, B01 and B06 and two on B08 (Figure 3b). Two associations, at SNP B01_43311767 (−log10 (*p*) = 7.51–10.34) and at SNP B08_24075810 (−log10 (*p*) = 6.02–7.68), were commonly detected by both models. The strongest association (−log10 (*p*) = 10.34) was at SNP B01_43311767 in BLINK. Compared to total GSLs and sinigrin, more SNPs were found to be significantly associated with gluconapin concentration. BLINK detected a total of 14 associated SNPs, comprising one SNP each on chromosomes A03, A06, A08, A10, B02, B03, B04 and B05, two on B01 and four on A02. FarmCPU returned six associated SNPs on chromosomes A02, A06, A08, B02, B03 and B05. These associations were distinct, although the association at SNP B02_48309648 in BLINK and SNP B02_48309753 in FarmCPU were only 105 bp apart. SNP B02_48309648 in BLINK represented the strongest association (−log10 (*p*) = 17.47). FarmCPU appeared the most suitable model for total GSLs and sinigrin with respect to the control of spurious associations as most observed *p*-values correlated with expected *p*-values, with only a few *p*-values deviating sharply at the tail end (Figure S2a,b). For gluconapin, BLINK associated a higher number of SNPs, while controlling best for spurious associations (Figure S2c). 

### 2.5. Significant GWAS Hits Had Known and Potential GSL Genes in Their Vicinity

The LD decay plot based on 14,125 SNPs suggested no effective LD (threshold of r^2^ = 0.1) at distances above 500 kb (Figure S4); hence, the search for potential candidate genes (using the *B. juncea* var. *tumida* V1.5 annotation) proximal to the trait-associated SNPs was limited to 250 kb upstream and downstream of the SNP position. Based on their homology with *Arabidopsis* genes and respective annotation, candidate genes were classified as known or potential GSL genes (Table 1). 

For total GSLs, homologues of two known GSL genes were identified near SNP A02_3567961, a significant SNP detected in BLINK and explaining around 7% of the observed trait variation (phenotypic variation explained—PVE). These were *GSTF11* [33,34,35] at 39.61 kb upstream and *SCPL17* [36] at 68.54 kb downstream. SNP A02_11235033, the most reliable association detected in all four models and accounting for 6% PVE, was located 128.81 kb upstream of a homologue of *OBP2*, encoding a known regulator of GSL biosynthesis [37]. SNP B02_7295738, which was detected in both FarmCPU and SUPER with 11% PVE, was found located near two potential GSL genes. Homologues to the potential GSL gene *amino acid permease 4* (*AAP4*) at 213.38 kb upstream and *SAL1* at 246.34 kb were found. SNP B08_66155255, detected only in FarmCPU, albeit at 37% PVE, was a genic SNP within a potential GSL gene, a putative *CYP18-3*. Moreover, at 17.56 kb, another potential GSL gene, a *putative 2-oxoglutarate-dependent dioxygenase* gene was found.

For sinigrin, SNP A03_27702263 with 4% PVE, detected by FarmCPU and SUPER, had homologues of several known GSL regulatory genes in proximity. These included a putative *MYB28* [38,39] at 118.32 kb upstream, as well as a putative *MYB34* [40,41] and a *MAM1* [42,43] homologue at 115.48 kb and 160.65 kb downstream. SNP B04_9016612 with 7% PVE, significant in BLINK, was found close to a homologue of the known GSL gene *FMO_GS-OX5_* [44,45] at 1.51 kb upstream. B04_17138489 with 12% PVE, which was significantly associated only in BLINK, was flanked by a potential GSL gene homologous to *phosphoserine aminotransferase 1* (*PSAT1*) at 12.75 kb. 

For gluconapin, SNP A02_34185026, detected by BLINK at 11% PVE, was found to be flanking an *LSU2* homologue, a potential GSL gene, at 5.75 kb downstream. SNP A02_34995417 with 1% PVE, detected in BLINK, was found to be located near additional homologues of *MYB28* and *MYB34* at 81.62 kb and 96.36 kb downstream, respectively. SNP A10_999168, solely detected by BLINK, was flanked by potential GSL genes monothiol glutaredoxin S11 (GRXS11) at 105.45 kb upstream and a UDP-glycosyltransferase 71C3 (UGT71C3) at 115.13 kb downstream. Variation within these two potential GSL genes may have contributed to the 11% PVE of this SNP. With 7% PVE, SNP B01_44925254, detected in BLINK and SUPER, was located near potential GSL genes *RETICULATA-RELATED 3* (*RER3*) at 105.45 kb upstream and a *Cysteine Synthase D1* (*CYSD1*) at 213.34 kb upstream. The strongest association from both BLINK and FarmCPU was only 105 bp apart and was considered the same association, SNP B02_48309648-753 with 3% PVE. This association was 180 kb upstream of a *HY5* homologue, encoding a known regulator of GSL biosynthesis [46]. SNP B03_474869, detected in FarmCPU with 6% PVE, was located near a potential GSL gene, *SULPHUR DEFICIENCY-INDUCED 2* (*SDI2*), at 23.76 kb. On the other hand, SNP B03_7408562, detected in BLINK and MLMM, was found to be near a potential GSL gene, *aldehyde dehydrogenase family 2 member B7* (*ALDH2B7)*, located at 135.05 kb downstream.

## 3. Discussion

### 3.1. Population Structure

The population structure of a diversity panel can confound GWA analysis through spurious associations [47]. Population clustering based on the UPGMA tree and ADMIXTURE (at K = 2) reflected a broad grouping based on geographical origin, with one group composed mainly of genotypes from South Asia (India and Pakistan) and another group from outside of South Asia (Figure 1a,b). Recent studies on population structure in Indian mustard reported a similar trend, with optimal Ks in the range of K = 2–3 [20,48]. In our study, ADMIXTURE K = 2 split the panel based on geographical origin (Figure 1b), with further sub-structuring of the two broad clusters until K = 4, a sensible K choice based on the cross-validation error (Figure 1c). The admixed samples may have resulted from interbreeding of the two population groups in variety improvement efforts. PCA was also concordant with the other methods (Figure 1d). The distribution of total GSLs, sinigrin and gluconapin in our diversity panel resembled that of a different panel of 190 accessions of diverse geographical origin, quantified for the same chemical traits [14] (Figure 2a–c). ADMIXTURE clustering reflected in the distribution of sinigrin and gluconapin confirmed previous reports on the correlations of GSL profiles with origin. Accessions from South Asian countries India and Pakistan (cluster 1) contained mostly gluconapin and lower levels of sinigrin, while accessions from outside of South Asia (cluster 2) mostly contained sinigrin in seeds [49,50,51,52]. This structure depicts crop divergence leading to these two varietal subgroups based on different end uses [18,53]. A strong selection for the health-beneficial GSL in East-European-type mustard for leafy vegetable and condiment cultivation was attributed to a predominant accumulation of sinigrin in samples originating from outside of South Asia. On the other hand, in India, cultivation was geared towards edible oil use, with yield and increasing the oil content as the main focus of selection for varietal improvement and not for a specific GSL type [18,51]. Thus, accessions from the Indian subcontinent, though predominantly accumulating gluconapin, also accumulated a lower proportion of sinigrin in our panel, consistent with earlier reports [9,50,51]. As such, the individual correlations of sinigrin and gluconapin with total GSLs were reflected more accurately at subgroup level than in the full panel (Figure 2f,g). In cluster 1, a weaker correlation between gluconapin and total GSLs reflected the presence of other GSLs in the total GSLs in these accessions. Conversely, in cluster 2, an almost perfect correlation was observed between sinigrin and total GSLs, attributed to higher homogeneity of the GSL profile. While this structure and the inter-trait correlations might have confounding effects on the GWAS, the resulting q-q plots for the two selected models suggested that these covariates were well accounted for and corrected in our analysis.

### 3.2. Candidate Genes Identified in the Vicinity of Associated SNPs

With the development of newer models with improved statistical power, GWAS recently incorporated multiple model approaches to maximise the power of QTL detection [25,54,55,56]. FarmCPU and BLINK are two of the newest models, with demonstrated superiority in statistical power compared to earlier GWAS methods [57,58]. The single SNP peaks observed from our GWAS using BLINK and FarmCPU were characteristic results for these models. Compared with other earlier models that display large peaks with multiple SNPs characteristic of “Manhattan” plots, these models highlight only the most significant marker in each association [54,57,58]. We located several strong homologues of known *Arabidopsis* GSL biosynthetic and regulatory genes, as well as potential GSL genes, in the vicinity of most of the significantly associated SNPs (Table 1). The majority of SNPs showed minor effects of around 10% PVE or less, as expected for a complex quantitative trait. An exception was SNP B08_66155255, with 37.03% PVE for total GSLs. These known and potential GSL genes were annotated as such in SuCCombase (https://plant-scc.org, accessed on 7 September 2021) [59], a curated repository of genes involved in the metabolism of sulphur-containing compounds including GSLs. While the “known” genes were listed in the inventory of GSL biosynthetic pathways in *Arabidopsis* [4,5], the “potential GSL genes” were identified from published co-expression data, which pinpoint genes that might be involved in GSL biosynthesis, yet lack experimental support.

Of the seven listed candidate genes for total GSL concentration, three were found on chromosome A02, two on B02 and two on B08 (Table 1). *BjuA041358* and *BjuA041338* were homologues of two GSL structural genes, *GSTF11* and *SCPL17*, respectively, and were linked to SNP A02_3567961. *GSTF11* encodes glutathione S-transferase F11, responsible for converting the intermediate derivative aci-nitro compounds to reduced glutathione (GSH) conjugates during aliphatic GSL core structure synthesis [33,34,35], making *BjuA041358* the stronger candidate. *SCPL17*, on the other hand, is involved in the production of benzoyloxy GSLs in *Arabidopsis* [36], making *BjuA041338* a less likely candidate. SNP A02_11235033 was a high-confidence association, considering that it was detected in all four models. The only candidate gene in this region was *BjuA045411*, a homologue of *OBP2* encoding a DNA-binding-with-one-finger (DOF) transcription factor [60], demonstrated to regulate indolic GSL in *Arabidopsis* [37]. Since nearly all GSLs in *B. juncea* are aliphatic, however, this *OBP2* homologue would need to have a divergent role to account for the total GSL variation. The association at SNP B02_7295738, the SNP with the second highest PVE (11%) for total GSLs, was linked to two potential GSL genes: *BjuB047551*, a homologue of *AAP4* encoding an amino acid permease 4, and *BjuB047557*, a homologue of *SAL1* encoding an inositol polyphosphate 1-phosphatase [59]. Given the high predicted peptide sequence similarity (94%), the *AAP4* homologue was likely the better candidate gene compared to the *SAL1* homologue at 65% similarity. Despite an exceptionally high PVE of 37.03% for total GSLs, no homologues to known GSL genes were found in the vicinity of SNP B08_66155255. However, SNP B08_66155255 was located within the gene model of *BjuB019211,* a homologue of *CYP18-3*, a putative peptidyl-prolyl cis-trans isomerase potentially involved in GSL metabolism, as suggested by co-expression with known GSL genes [59]. Furthermore, around 18 kb upstream, a probable 2-oxoglutarate-dependent dioxygenase encoding gene was located. Known GSL genes *AOP2* and *AOP3* similarly encode 2-oxoglutarate-dependent dioxygenases, which catalyse the side-chain oxygenation in the aliphatic GSL core synthesis [61,62]. The high PVE of SNP B08_66155255 merits further investigation.

Of the five candidate genes associated with sinigrin, homologues of three known GSL regulatory genes were found in the vicinity of SNP A03_27702263. *BjuA042263*, *BjuA042229* and *BjuA042223* were homologues of *MYB28*, *MYB34* and *MAM1*, respectively. *MYB28*, also known as *HAG1* (*HIGH ALIPHATIC GLUCOSINOLATE 1*), positively regulates aliphatic GSLs [38,39], with gain-of-function and knock-down mutants showing contrasting levels of aliphatic GSLs and transcript levels of corresponding biosynthetic genes [38]. *MYB28* was further identified and validated through combined multi-omics approaches, including GWAS, as the major gene controlling leaf and seed GSL content in *B. napus* [25], suggesting that natural variation at this locus drives phenotypic variation. In oilseed *B. juncea*, targeted silencing of a *MYB28* orthologue led to the down-regulation of GSL biosynthesis [6], making *BjuA042263* a very strong candidate for this QTL region and a high priority for our further validation efforts. On the other hand, *MYB34* mainly exerts its role in the roots to regulate indolic GSL synthesis [40,41] and *MAM1* is a methylthioalkylmalate synthase involved in the GSL side-chain elongation of short-chained aliphatic GSLs [42,43], suggesting their respective *B. juncea* homologues to be less likely causal for the effects associated with SNP A03_27702263. SNP B04_9016612, with 7% PVE, was a genic SNP within *BjuB028146*, a homologue of *FMO_GS-OX5_* encoding a flavin-containing monooxygenase. *FMO_GS-OX5_* functions in aliphatic GSL side-chain modification by S-oxygenation of the basic aliphatic GSL derivatives [44,45], making *BjuB028146* a high-priority candidate gene. *BjuB028703*, homologous to the potential GSL gene *PSAT1*, was located near SNP B04_17138489, with 12% PVE. *PSAT* encodes a putative phosphoserine aminotransferase in the serine biosynthetic pathway [63]. Although this locus had a high PVE, *PSAT1* has not been directly associated with aliphatic GSL metabolism. However, serine is a substrate for tryptophan biosynthesis, which in turn is a precursor for the production of indolic GSLs [64]. Furthermore, in *Arabidopsis*, it is regulated by *MYB34* and *MYB51*, two activators of indolic GSL biosynthesis [63].

Ten candidate genes, three known and seven potential GSL genes, can be speculated to contribute to gluconapin variation. Among these, *BjuA033112* a homologue of *LSU2 (RESPONSE TO LOW SULPHUR 2)*, was found less than 6 kb from SNP A02_34185026. While LSU proteins are of unknown function, they were demonstrated to be important stress-related hubs [65] and considered marker genes of sulphur metabolism [66], making *BjuA033112* a good candidate to account for the considerable 11% PVE of this locus. Interestingly, *MYB28* and *MYB34* homologues, additional copies of which were already implicated in the variation in sinigrin concentration on chromosome A03, were found in the vicinity of SNP A02_34995417, although this SNP contributed little to the observed gluconapin variation. *BjuA002140* was a homologue of *MYB28*, while *BjuA001524* was a homologue of *MYB34*. Copy number variation (CNV) of *MYB28* homologues on different chromosomes might have led to the divergence that specifically accounts for sinigrin and gluconapin accumulation in different genetic backgrounds. Recently, CNV was uncovered on *MYB28* loci through pairwise sequencing of a vegetable variety, T84-66, and an Australian oilseed variety, AU213 [17]. Among the associations with high PVE (11%) was SNP A10_999168, located near homologues of two potential GSL genes. *BjuA037341* was a homologue of *UGT71C3* encoding an UDP-glycosyltransferase, and *BjuA037371* a homologue of *GRXS11* encoding monothiol glutaredoxin, implicated in nitrogen signalling [67]. The direct involvement of UDP-glycosyltransferase *UGT74B1* [68] and of *UGT74C1* in aliphatic GSL core synthesis [69] suggests that *BjuA037341* is the higher-confidence candidate for this association. Having been detected in BLINK and SUPER, SNP B01_44925254 was a reliable and strong association (−log10 (*p*) = 16.86) for gluconapin. However, homologues of only two potential GSL genes were found in proximity. These were *BjuB006588*, homologous to *RER3* encoding *RETICULATA-RELATED 3*, and *BjuB006607,* homologous to *CYSD1*, a cysteine synthase and a member of the O-acetylserine(thiol)lyase (*OASTL*) gene family. *OASTLs* include *OASA1*, an S assimilation pathway gene that catalyses the biosynthesis of cysteine and a precursor for GSL formation [70].

A *LONG HYPOCOTYL 5* (*HY5*) homologue, *BjuB009816*, was located near the high-confidence gluconapin associations SNP B02_48309748-53 at a PVE of 3%. *HY5*, a transcription regulator, was shown to partly control the light regulation of GSL biosynthetic genes, as well as many genes in the sulphate assimilation pathway [46]. Additionally, *hy5 Arabidopsis* mutants showed altered expression of GSL biosynthetic genes and MYB TFs associated with aliphatic GSL regulation [46]. *BjuB005751*, a homologue of another potential GSL gene, *SDI2* encoding SULPHUR DEFICIENCY-INDUCED 2, was located near SNP B03_474869. Under sulphur-limiting conditions in *Arabidopsis*, SDI2 acts as a repressor of aliphatic GSL biosynthesis at transcript and metabolite levels [71]. Despite being detected under non-limiting sulphur conditions, this *B. juncea SDI2* homologue could affect GSL composition. Lastly, *BjuB003011* a homologue of a potential GSL gene *ALDH2B7* encoding an aldehyde dehydrogenase family 2 protein, was located near SNP B03_7408562. While two models detected this association for gluconapin, no literature support was found for the involvement of *ALDH2B7* in GSL biosynthesis, aside from it being listed as a potential GSL gene in SuCCombase [4,59].

We found no overlap in proposed candidate genes with the GWAS study by Akhatar et al., 2020, probably owing to different aims, translating to differences in panel composition, different methods of GSL quantification and differences in cultivation. Furthermore, they limited the candidate gene search to a narrow window of 25 kb upstream and downstream of peak SNPs. The Akhatar et al., 2020, study was conducted under field conditions, with the aim to study the effects of various nitrogen levels. They used only 92 accessions, which were phenotyped for GSL content using Near-Infrared Reflectance Spectroscopy (NIRS) on intact seeds to predict total GSLs. In contrast, we phenotyped a larger, more diverse panel grown under controlled conditions, using quantitative approaches for several specific GSLs. Their study detected associations using a relaxed −log10 (*p*) ≥ 3 threshold and proposed proximate candidate genes encoding for shikimate kinases (chromosome A04), chorismate mutase (chromosomes A06 and B04), jasmonate O-methyltransferase (chromosome B03), branched-chain-amino-acid transaminase (chromosome B06), cytochrome P450 enzyme CYP81G1 (chromosome B06) and MYB44 transcription factor (chromosome B06). Of these candidates, only the *CYP81G1* was listed as a potential GSL potential gene in SuCCombase and no genes had homologues of known and validated function in GSL biosynthesis or regulation. In contrast, our GWAS study used a controlled-environment growing condition, coupled with HPLC-MS-based analysis for the accurate quantification of individual GSLs, and applied a stringent Bonferroni threshold for the detection of associations. Yang et al., 2021, identified only two major control loci in a panel of 183 mixed vegetable and oilseed accessions phenotyped for individual GSLs using HPLC and genotyped at a density of 689,411 SNPs. *MYB28* (chromosome A02 and A09) was highlighted as a priority candidate gene, supporting the role of *MYB28* as a key regulator of GSL accumulation in *B. juncea*. Thus, our findings add value to previous studies and provide an exceptional resource of novel candidate gene homologues to known structural and regulatory genes of GSL metabolism. Further validation through allele mining and gene expression profiling is warranted, especially for associations explaining high levels of phenotypic variation and detected in multiple models.

## 4. Materials and Methods

### 4.1. Plant Materials and Growing Conditions

A diversity panel of 158 Indian mustard accessions from 28 countries, which had undergone two rounds of single seed descent (SSD) (Table S1), were grown in a CONVIRON^®^ plant growth chamber (model: PGCFLEX, Winnipeg, MB, Canada) at Southern Cross University Lismore, New South Wales (28.8° S, 153.3° E), from March to mid-May 2020. Several seeds per accession were sown at 5 mm depth in a 10-cm-diameter free-draining plastic pot filled with commercial potting soil and thinned to one plant per pot two weeks after emergence. Each accession was grown in triplicate in a complete randomised block design. Three-week-old seedlings were supplied with 25 mL of diluted to half strength liquid fertiliser Canna A + B (CANNA Australasia, Subiaco Western Australia, delivered through syringe plunger, per pot. The growing conditions were set at 16 h of artificial lighting at 22 °C and eight hours of dark at 16 °C. Harvesting was done when all siliques were dried, and harvested siliques were further air-dried at 40 °C for 72 h before threshing.

### 4.2. Glucosinolate Analysis

In total, three biological replicates per accession (consisting of two individual seeds each) were used for quantifying GSL concentrations, following the method by Borpatragohain et al., 2019 [72]. In brief, two seeds per sample were placed in an Eppendorf safe-lock tube, to which 1.5 mL of 70% methanol and a 5 mm stainless-steel bead was added. The samples were then homogenised using a Qiagen Retsch MM 301 TissueLyser II (Qiagen Retsch, Hilden, Germany)) at 30 Hz for 45 s. Next, the samples were centrifuged for 15 min at 15,000 rpm at 7 °C using a Sigma laboratory tabletop centrifuge (Osterode am Harz Germany). An aliquot of 200 µL was transferred from the supernatant solution after centrifugation to a 2 mL Agilent HPLC screw-cap vial. The samples were then dried down using Martin Christ Alpha RVC (Osterode am Harz Germany) at successively reduced pressure of 180, 120, 80, 50, 20 and 5 mbar each at one-hour intervals, while 5 mbar was kept overnight. The dried samples were resuspended in 1.5 mL water containing 1.17 µmol mL^−1^ glucotropaeolin (a GSL not found in Brassicas) as internal standard. The tubes were mixed by inverting several times. Eight individual GSLs were quantified, including sinigrin (SIN), gluconapin (GNP), progroitrin (PGT), epi-pogroitrin (EPI), glucoiberin (GIB), glucoraphanin (GRF), glucobrassicin (GBS) and gluconarturtiin (GNT), using an Agilent 1260 Infinity II High Performance LC-MS instrument (Agilent Technologies, Palo Alto, CA, USA). HPLC-MS parameters used are detailed in Supplemental File S3. Total GSLs is the sum of the eight GSLs measured.

### 4.3. Bioinformatic Analyses and Data Processing

Illumina’s FastQ sequence outputs were demultiplexed using Axe [73]. Both reads from the paired-end data were aligned against the *B. juncea* var. *tumida* T84-66 V1.5 genome reference (http://39.100.233.196:82/download_genome/Brassica_Genome_data/Braju_tum_V1.5, accessed on 15 January 2022) [53]. SNP calling was carried out using the Stacks pipeline [74], using default parameters and a low-level filter by looking for a minimum allele frequency of 5% for an SNP to be considered. Among the duplicated samples, the sample with the lower call rate was removed. Filtering of the resulting variant table for SNPs with a 60% call rate, non-minor allele frequency filtered and 10% maximum marker heterozygosity was done using TASSEL 5.2.73 [75]. Missing marker states for all remaining unique genotypes were imputed using Beagle 5.2 [76] with default parameters and the effective population size (Ne) set to 500,000.

### 4.4. Statistical Analysis

Residual distribution and quantile–quantile plots were visualised using Genstat 64-bit Release 18.1 (VSN International Ltd., Hemel Hempstead, England UK) to assess the normality and homoscedasticity of the phenotype data. Data were log10 (x + 0.01) transformed for subsequent estimation of the variance components and heritability values using REML Restricted Maximum Likelihood (REML) implemented in Genstat 64, as well as input for GWA. Best Linear Unbiased Predictions (BLUPs), calculated using genotype and replicate effects in REML, were used as phenotype input in GWAS. Correlations among GSL traits using raw mean values were computed using the ‘ggpubr’ package [77], implemented in the R environment.

### 4.5. Genome-Wide Association Analysis

Marker–trait association was performed using the Genome Association and Prediction Integrated Tool (GAPIT Version 3) [78,79]. To select the best models, an initial analysis using the four most recommended models as discussed in the GAPIT manual based on statistical power was conducted [79]. These were multiple locus mixed linear model (MLMM) [80], Settlement of MLM Under Progressively Exclusive Relationship (SUPER) [81], Fixed and random model circulating probability unification (FarmCPU) [57], Bayesian-information and Linkage-disequilibrium Iteratively Nested Keyway (BLINK) [58]. The best models were selected based on the resulting q-q plots, which reflected how well each model accounted for population structure and familial relatedness. Manhattan plots were visualised using R package ‘CMplot’ [82].

### 4.6. Cluster, Population Structure and Principal Components Analysis

A separate set of 1174 higher-confidence SNPs imputed and filtered for >80% call rate, 5% minor allele frequency (maf) and 10% maximum heterozygosity, covering pseudochromosomes, and linkage-disequilibrium (LD) pruned, was used for cluster, population and principal components analyses. LD pruning was done using Plink [83] (version 1.07) with the following parameters: window of 50 SNPs, step size of five markers and an r^2^ threshold of 0.4 [84]. An UPGMA (unweighted pair group method with arithmetic mean) tree was built for cluster analysis of all 158 lines. The genetic distance input for tree building was simple matching coefficients calculated in TASSEL (version 5.2.72) [75] and UPGMA was visualised using ITOLv6 (https://itol.embl.de/, accessed on 3 September 2021). A maximum likelihood estimate for population structure was carried out in ADMIXTURE [85] and barplots for Q matrix (probability of group membership) were visualised using package ‘pophelper’ [86] implemented in the R environment. The analysis was done for K = 1 to K = 10, and a ten-fold cross-validation procedure was used to determine the “best” K. PCA was conducted in TASSEL (version 5.2.72) and plotted using the ‘ggplot2′ [87] R package.

### 4.7. Candidate Genes within Significant SNPs

Predicted candidate genes within 250 kb upstream and downstream of each significantly associated SNP were identified using the *B. juncea* BRAD v.1.5 annotation. The BRAD V1.5 genes were annotated for putative function by alignment to the *Arabidopsis* TAIR10 release using NCBI BLASTP [88,89], integrated into the in-house SCPS Galaxy (http://lr-scps5-rh7v.scps.scu.edu.au:8080, accessed on 9 September 2021), and associating the annotation of the *Arabidopsis* genes in the top-scoring hits. All these annotations and genome information were integrated into the SCPS Galaxy. Next, we matched the *Arabidopsis* locus identifiers from our BLAST+ list and that of “known” and “potential GSL genes” curated in SuCCombase (https://plant-scc.org, accessed on 7 September 2021) for listing our candidate genes. Top hits identified as either “known” or “potential GSL genes” based on SuCCombase were prioritised as candidate genes. In a few cases, we chose the “known” or “potential GSL gene” even if they ranked second to third in BLASTP, provided that the percent identity was more than 60% across more than 50% of the total length alignment.

## Figures and Tables

**Table 1:** Peak SNP characteristics and respective candidate genes homologous to known and potential *Arabidopsis* genes involved in GSL metabolism.

Trait ^a^	Peak SNP	*p*-Value	PVE ^b^	Model ^c^	Candidate Gene	Homologous Gene in *Arabidopsis*	% Amino Acid Identity	Distance to Peak SNP [kb]	*Arabidopsis* ID	Gene Description
TGSL	A02_3567961	5.08 × 10^−8^	6.63	B	BjuA041358	*GSTF11* [33,34,35]	69.16	−39.61	AT3G03190.1	GSL core structure synthesis
					BjuA041338	*SCPL17* [36]	61.43	68.54	AT3G12203.3	GSL side-chain modification
	A02_11235033	2.54 × 10^−7^	5.83	B, F, M, S	BjuA045411	*OBP2* [37]	84.92	−128.81	AT1G07640.1	GSL regulation
	B02_7295738	4.09 × 10^−10^	11.41	F, S	BjuB047551	*AAP4*	94.21	213.38	AT5G63850.1	potential GSL gene
					BjuB047557	*SAL1*	64.76	246.34	AT5G63980.1	potential GSL gene
	B08_66155255	1.90 × 10^−7^	37.03	F	BjuB019211	*CYP18-3*	65.48	−0.7	AT4G38740.1	potential GSL gene
					BjuB019215	*Probable 2-ODD* ^d^	61.77	−17.56	AT5G05600.1	potential GSL gene
SIN	A03_27702263	1.50 × 10^−7^	3.76	F, S	BjuA042263	*MYB28* [38,39]	79.95	−118.32	AT5G61420.2	GSL regulation
					BjuA042229	*MYB34* [40,41]	71.32	115.48	AT5G60890.1	GSL regulation
					BjuA042223	*MAM1* [42,43]	82.72	160.65	AT5G23010.3	GSL side-chain elongation
	B04_9016612	5.04 × 10^−6^	6.84	B	BjuB028146	*FMO_GS-OX5_* [44,45]	69.41	−1.51	AT1G12140.3	GSL side-chain modification
	B04_17138489	2.51 × 10^−6^	11.71	B	BjuB028703	*PSAT1*	83.57	12.75	AT4G35630.1	potential GSL gene
GNP	A02_34185026	1.64 × 10^−7^	11.24	B	BjuA033112	*LSU2*	86.022	5.75	AT5G24660.1	potential GSL gene
	A02_34995417	1.29 × 10^−6^	0.72	B	BjuA002140	*MYB28* [38,39]	67.46	81.62	AT5G61420.1	GSL regulation
					BjuA001524	*MYB34* [40,41]	72.00	96.36	AT5G60890.1	GSL regulation
	A10_999168	6.85 × 10^−7^	10.72	B	BjuA037371	*GRXS11*	96.97	−105.45	AT1G06830.1	potential GSL gene
					BjuA037341	*UGT71C3*	79.19	115.13	AT1G07260.1	potential GSL gene
	B01_44925254	1.38 × 10^−17^	7.15	B, S	BjuB006588	*RER3*	78.44	−105.02	AT3G08640.1	potential GSL gene
					BjuB006607	*CYSD1*	73.27	−213.34	AT3G04940.2	potential GSL gene
	B02_48309648-753	3.35 × 10^−18^	2.80	B, F	BjuB009816	*HY5* [46]	89.94	180.71	AT5G11260.1	GSL regulation
	B03_474869	2.49 × 10^−6^	6.03	F	BjuB005751	*SDI2*	82.50	23.76	AT1G04770.1	potential GSL gene
	B03_7408562	7.07 × 10^−8^	4.78	B, M	BjuB003011	*ALDH2B7*	91.01	135.05	AT1G23800.1	potential GSL gene

^a^ TGSL (Total GSLs), SIN (sinigrin), GNP (gluconapin). ^b^ Phenotypic variance explained by marker calculated in GAPIT. In case of co-detection with BLINK, PVEs obtained were from BLINK; if otherwise, PVEs were from FarmCPU. ^c^ BLINK(B), FarmCPU (F), MLMM (M), SUPER (S). ^d^ Probable 2-oxoglutarate-dependent dioxygenase.

## Data Availability

All relevant research data is included as supplementary material. The genotype data can be accessed through the following DOI: DOI 10.25918/data.186.

## Supplementary Materials

The following supporting information can be downloaded at: https://www.mdpi.com/article/10.3390/plants11030364/s1, Supplemental File S1. Glucosinolate measurements of the accessions in the diversity panel; Table S1. List of accessions and country of origin in the diversity panel; Figure S1. Residual distribution and normal plots for total GSLs, sinigrin and gluconapin; Table S2. Variance components analysis and descriptive statistics for total GSLs, sinigrin and gluconapin evaluated in 158 diverse *B. juncea* L. accessions; Figure S2. Correlations of major GSLs, sinigrin and gluconapin, and total GSLs in ADMIXTURE clusters; Figure S3. Quantile–quantile plots reflecting correspondence between observed and expected −log10 (*p*) values from association analyses using four models (SUPER, MLMM, FarmCPU, BLINK) for total GSLs, sinigrin and gluconapin; Supplemental File S2. List of SNPs passing the Bonferroni threshold from four models; Figure S4. Linkage disequilibrium (LD) depicted based on squared correlation coefficient of pairwise markers in a sliding window of 100 SNP markers; Supplemental File S3. HPLC-MS parameters used for glucosinolate analysis.
