# Supplementary material for: Genome-Wide Association Reveals Trait Loci for Seed Glucosinolate Accumulation in Indian Mustard (Brassica juncea L.)
Source: Plants (Basel). 2022 Jan 28;11(3):364. doi: 10.3390/plants11030364 (PMC8838242; doi:10.3390/plants11030364)
Supplement: Supplementary file 1 [file plants-11-00364-s001.zip › Figure S2.pdf]

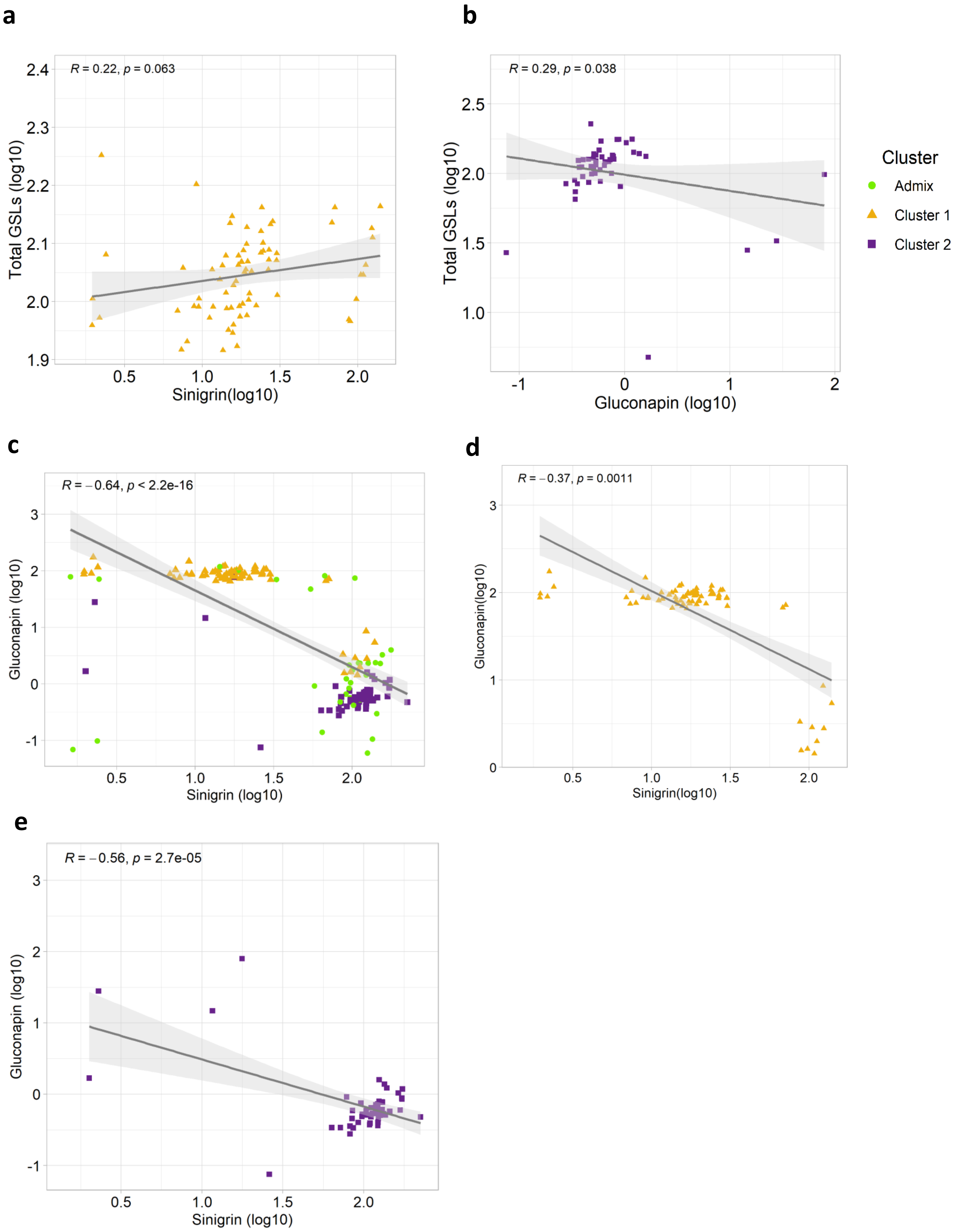

Figure S2. Correlations of major GSLs sinigrin and gluconapin, and total GSLs in ADMIXTURE clusters. **a**) Correlation of log-transformed values of sinigrin and total GSLs in cluster 1.; **b**) Correlation of log-transformed values of gluconapin and total GSLs in cluster 2.; Correlations log-transformed values of sinigrin and gluconapin in: **c**) full panel, **d**) cluster 1 and **e**) cluster 2
