# Supplementary material for: Genome-Wide Association Reveals Trait Loci for Seed Glucosinolate Accumulation in Indian Mustard (Brassica juncea L.)
Source: Plants (Basel). 2022 Jan 28;11(3):364. doi: 10.3390/plants11030364 (PMC8838242; doi:10.3390/plants11030364)
Supplement: Supplementary file 1 [file plants-11-00364-s001.zip › Figure S3.pdf]

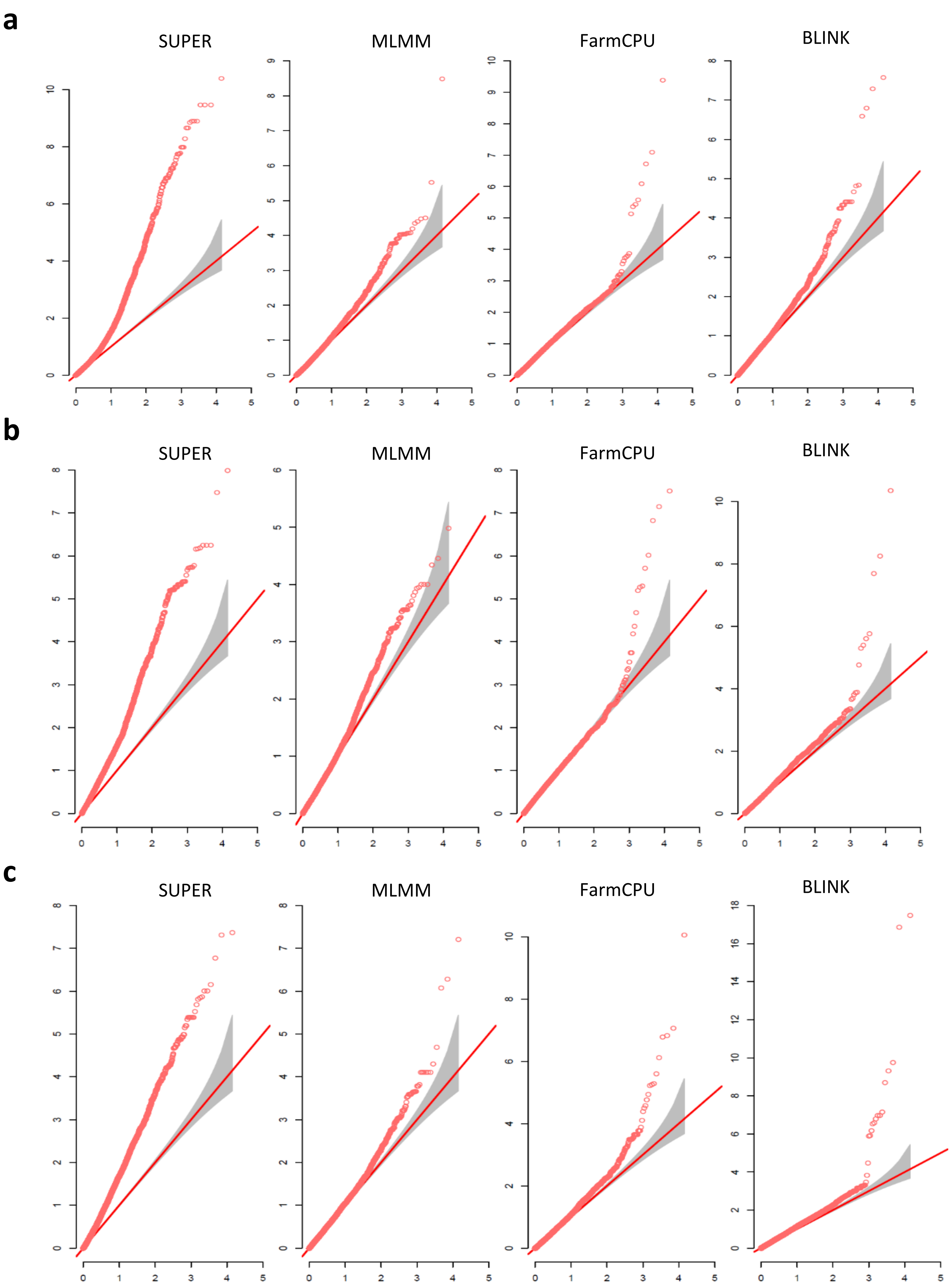

Figure S3. Quantile-quantile plots reflecting correspondence between observed and expected $-\log_{10}(p)$ values from association analyses using four models (SUPER, MLMM, FarmCPU, BLINK) for: **a**) total GSL; **b**) sinigrin and; **c**) gluconapin.
