# Supplementary material for: Genome-Wide Association Reveals Trait Loci for Seed Glucosinolate Accumulation in Indian Mustard (Brassica juncea L.)
Source: Plants (Basel). 2022 Jan 28;11(3):364. doi: 10.3390/plants11030364 (PMC8838242; doi:10.3390/plants11030364)
Supplement: Supplementary file 1 [file plants-11-00364-s001.zip › FIgure S4.pdf]

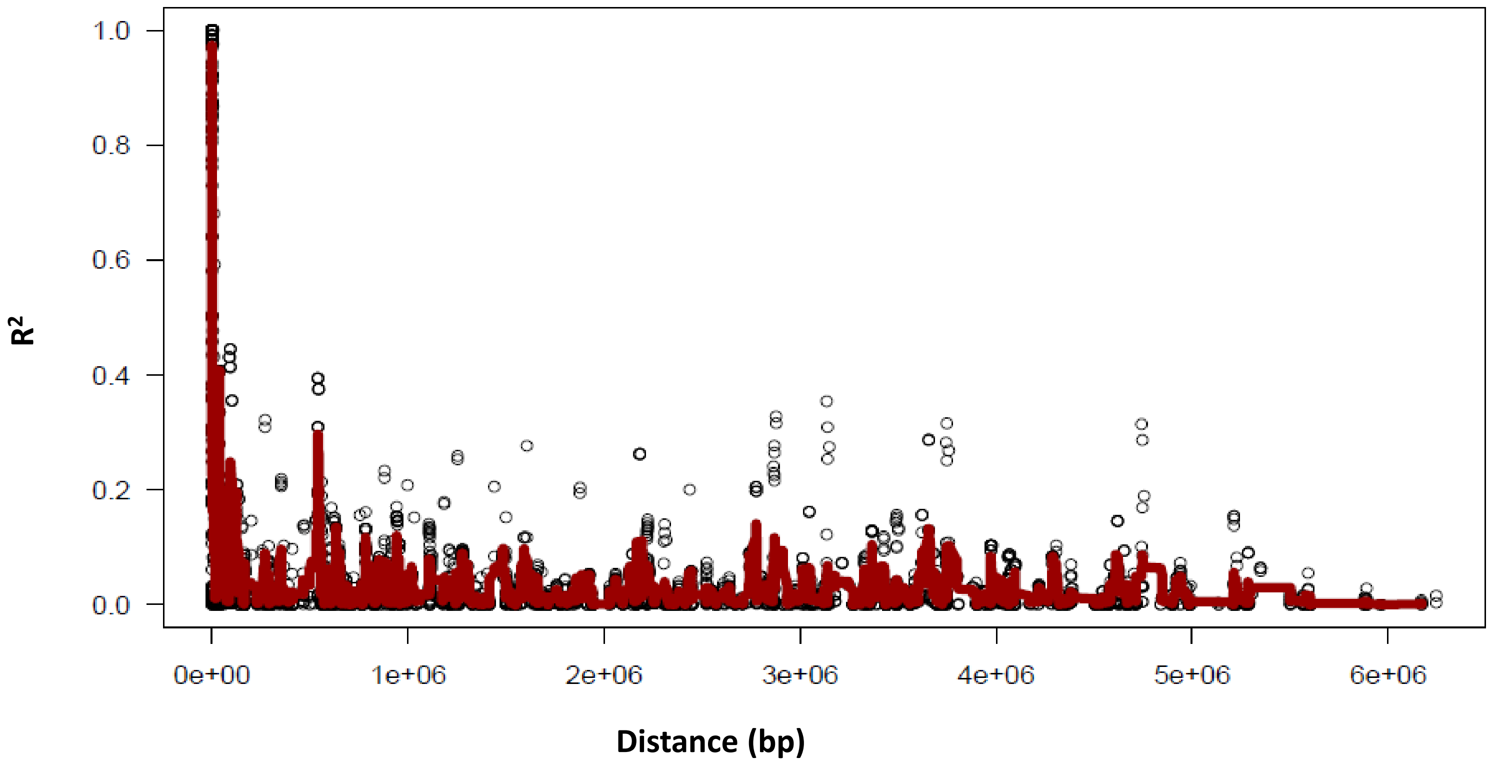

Figure S4. Linkage disequilibrium (LD) depicted based on squared correlation coefficient of pairwise markers in a sliding window of 100 SNP markers. Red line represents moving average of 10 adjacent markers.
