# Supplementary material for: Genome-Wide Association Reveals Trait Loci for Seed Glucosinolate Accumulation in Indian Mustard (Brassica juncea L.)
Source: Plants (Basel). 2022 Jan 28;11(3):364. doi: 10.3390/plants11030364 (PMC8838242; doi:10.3390/plants11030364)
Supplement: Supplementary file 1 [file plants-11-00364-s001.zip › Supplemental File S3.pdf]

Supplemental File S3. HPLC-MS parameters used for glucosinolate analysis

All extracts were analyzed using an Agilent 1260 Infinity II High Performance LC-MS instrument (Agilent Technologies, Palo Alto, CA, USA) equipped with an autoinjector, vacuum degasser, binary pump and diode array detector (DAD, 1260), coupled with an Agilent InfinityLab LC/MSD XT single quadrupole mass analyser. A Kinetex® 2.6 µm EVO C18 reverse phase column (100 x 2.1 mm internal diameter) (Phenomenex, Torrance, CA, USA) was used, with temperature set at 30°C. A linear gradient elution program was applied consisting of a mobile phase containing Milli-Q water with 0.01% trifluoroacetic acid (TFA) (solvent A) and acetonitrile with 0.005% TFA (solvent B) at a flow rate of 0.3 mL/min and 5µL injection volume. The 10 min run consisted of 0% B (4 min), 25% B (6.10 min), 100% B (6.20 min) and 0% B (10 min). The mass analyser was operated in atmospheric pressure ionization-electrospray (API-ES) mode with the following parameters: fragmentor, 150; capillary voltage, 3000 V (negative); drying gas flow, 12 L/min (N_2); vaporizer temperature, 350 °C; nebulizer pressure, 35 psi; drying gas temperature, 350 °C. Absorbance was monitored at 210, 280 and 360 nm. Single ion monitoring (SIM) mode was set to detect 7 ions simultaneously in negative ion mode using four available mass selective detection signal channels such as signal 1: sinigrin (SIN) at *m/z* ratio of 358 for 0-8 min and glucotropaeolin (GTP) at *m/z* ratio of 408 for 8 to 18 min, signal2: progoitrin (PGT) and epiprogoitrin (EPI) at *m/z* ratio of 388 for 0-18 min, signal 3: glucoiberin (GIB) at *m/z* ratio of 422 for 0-10 min and gluconasturtiin (GNT) at m/z ratio of 422 for 10-18 min, signal 4: gluconapin (GNP) at *m/z* ratio of 372 for 0-18 min. Glucotropaeolin (GTP), not found in brassicas, was used as the internal standard to monitor the performance of MS. All LC-MS settings and parameters above were optimized based on the manufacturer's recommendations and a number of flow injection experiments. All the organic solvents used in the analysis were HPLC or LC-MS grade. Commercial GSL standards were obtained from PhytoLab GmbH & Co. KG, Germany.
