# Supplementary material for: Genome-Wide Association Reveals Trait Loci for Seed Glucosinolate Accumulation in Indian Mustard (Brassica juncea L.)
Source: Plants (Basel). 2022 Jan 28;11(3):364. doi: 10.3390/plants11030364 (PMC8838242; doi:10.3390/plants11030364)
Supplement: Supplementary file 1 [file plants-11-00364-s001.zip › Table S1.pdf]

Table S1. List of accessions and country of origin in the diversity panel.

Number	Genotype Code	Name	Country of Origin
1	BJ85	887-TT3	Unknown
2	BJ2	Basanti	India
3	BJ9	Narendra swarna rai-9	India
4	BJ36	PBR92	India
5	BJ109	JM1	India
6	BJ184	JM-2	India
7	BJ42	RH8113	India
8	BJ4	Sej2	India
9	BJ59	Mustard seed brown	Unknown
10	BJ91	Vaibhav	India
11	BJ177	Rohini	India
12	BJ100	NDR8502	India
13	BJ41	RLM619	India
14	BJ32	P-36	Pakistan
15	BJ160	P-15	Pakistan
16	BJ156	MPIR	China
17	BJ132	Xinyou8	China
18	BJ168	XINYOU	China
19	BJ3	Xinyou9	China
20	BJ20	HanZhong Goa youCai	China
21	BJ33	Kranti	India
22	BJ146	RGN13	India
23	BJ176	Ashirwad	India
24	BJ54	RH781	India
25	BJ37	CPI104436	China
26	BJ141	CS-52	India
27	BJ96	cs-54	India
28	BJ29	Xinyou5	China
29	BJ165	Sanjucta	India
30	BJ65	Seeta	India
31	BJ48	PCR7	India
32	BJ116	RH819	India
33	BJ44	Laxmi	India
34	BJ133	Geeta	India
35	BJ66	ORI	Pakistan
36	BJ162	MRS8867	Afghanistan
37	BJ183	Jubilejnaja	Australia
38	BJ30	MRS88245	Bangladesh
39	BJ27	Rai14	Bangladesh
40	BJ7	Sampad	Bangladesh
41	BJ63	MRS88 331	Belgium
42	BJ128	MRS88396	Bhutan
43	BJ173	MRS902-14	Bhutan
44	BJ52	Domo	Canada
45	BJ129	Elka	Canada

46	BJ143	volgogradskaja	Canada
47	BJ39	PI257240	China
48	BJ157	Qu xi ai ke gai la	China
49	BJ73	MRS88 309	Czech Republic
50	BJ35	R 2458	Egypt
51	BJ178	Juncea Aus Aeth	Ethiopia
52	BJ95	MRS8861	Ethiopia
53	BJ158	Ranniana	Former Soviet Union
54	BJ150	MRS88328	Germany
55	BJ70	Kai choi	Hong Kong
56	BJ163	coss var long bailey	Hungary
57	BJ74	21709	India
58	BJ58	app mutant	India
59	BJ111	CSIRO62005	India
60	BJ125	CSR37	India
61	BJ68	CSR75	India
62	BJ179	IB1698	India
63	BJ108	IB709	India
64	BJ83	MRS88 23	India
65	BJ79	MRS88 285	India
66	BJ110	MRS88 356	India
67	BJ180	MRS88356	India
68	BJ104	MRS88 38	India
69	BJ124	PI179855	India
70	BJ159	PI180417	India
71	BJ112	PI 212970	India
72	BJ114	PI370744	India
73	BJ67	Prakesh	India
74	BJ123	RH30A	India
75	BJ75	RH7515	India
76	BJ92	Sarson	India
77	BJ181	SILIGURI CL-1	India
78	BJ38	Varuna	India
79	BJ78	common green	Japan
80	BJ28	Ki Karashina	Japan
81	BJ17	Kumamoto karashina	Japan
82	BJ12	Kai choiJ	Malaysia
83	BJ72	Samni	Malaysia
84	BJ174	MRS90-024	Maldives
85	BJ154	MRS90-009	Mexico
86	BJ127	MRS88367	Nepal
87	BJ57	MRS88367	Nepal
88	BJ139	MRS8892	Nepal
89	BJ16	MRS88 92	Nepal
90	BJ107	PI175067	Nepal
91	BJ1	1007-14	Pakistan
92	BJ61	35421	Pakistan
93	BJ51	K336	Pakistan

94	BJ15	K399	Pakistan
95	BJ144	K739	Pakistan
96	BJ115	K955	Pakistan
97	BJ164	LL-84	Pakistan
98	BJ26	MR588 81	Pakistan
99	BJ122	MRS90-007	Pakistan
100	BJ43	PI181026	Pakistan
101	BJ46	PI250130	Pakistan
102	BJ22	PI426326	Pakistan
103	BJ89	PI 426401	Pakistan
104	BJ55	QA-106	Pakistan
105	BJ71	Raya anmol	Pakistan
106	BJ50	S220	Pakistan
107	BJ45	S300	Pakistan
108	BJ64	SIN-31-73	Pakistan
109	BJ47	289	Poland
110	BJ152	Malopolska	Poland
111	BJ11	MRS88 343	Poland
112	BJ53	zeltesemiannaja230	Russian Federation
113	BJ153	Stalingrad	Sweden
114	BJ25	PI 249555	Thailand
115	BJ169	PI249555	Thailand
116	BJ149	MRS88 21	Turkey
117	BJ86	PI120923	Turkey
118	BJ5	PI169085	Turkey
119	BJ155	J/805/9	United Kingdom
120	BJ49	MRS88329	United Kingdom
121	BJ119	PI184290	United Kingdom
122	BJ161	Early yellow	United States
123	BJ118	Orient yellow	United States
124	BJ94	BHE	Unknown
125	BJ60	Juzanka	Unknown
126	BJ69	UCD-9	Unknown
127	BJ10	Vulcan	Unknown
128	BJ130	ZEM-2	Unknown
129	BJ138	Zero Erucic	Unknown
130	BJ142	Zero Erucic skorospelka	Unknown
131	BJ148	Zollerngold	Unknown
132	BJ40	BRA434/79	Yugoslavia
133	BJ137	Bingu	Zimbabwe
134	BJ113	NDAKupuka	Zimbabwe
135	BJ140	Tsunga	Zimbabwe
136	BJ62	Murasaki takana	Japan
137	BJ56	W1	India
138	BJ88	W3	India
139	BJ172	W9	India
140	BJ18	Janapechey	Bhutan
141	BJ14	Chothma	Bhutan

142	BJ34	Tsatsai	China
143	BJ31	Muliceps	China
144	BJ13	Xue Li Hong	China
145	BJ19	Siromo	Australia
146	BJ101	Pacific Gold-AUS	Australia
147	BJ102	Forge	United Kingdom
148	BJ24	Pacific Gold-USA	USA
149	BJ105	Muscon	Australia
150	BJ134	Lethbridge	Canada
151	BJ136	352	Australia
152	BJ93	Zero Erucic Lethbridge	Unknown
153	BJ8	C671	Canada
154	BJ82	JN28	Unknown
155	BJ131	Early Zero Erucic Lethbridge	Unknown
156	BJ175	99Y	Australia
157	BJ84	Cutlass	Canada
158	BJ6	Burgonde	Canada
