# Supplementary material for: Genome-Wide Association Reveals Trait Loci for Seed Glucosinolate Accumulation in Indian Mustard (Brassica juncea L.)
Source: Plants (Basel). 2022 Jan 28;11(3):364. doi: 10.3390/plants11030364 (PMC8838242; doi:10.3390/plants11030364)
Supplement: Supplementary file 1 [file plants-11-00364-s001.zip › Table S2.pdf]

Table S2. Variance components analysis and descriptive statistics for total GSLs, sinigrin and gluconapin evaluated in 158 diverse *B. juncea* L. accessions.

	Total GSLs	Sinigrin	Gluconapin
Variance components			
δ^2_g (SE)	0.03581 (0.004203	0.29362 (0.03280)	1.16033 (0.12959)
δ^2_{gr} (SE)	0.00192 (0.00038)	0.00225 (0.00048)	0.01225 (0.00181)
Residual	0.00315 (0.00021)	0.00416 (0.00283)	0.00906 (0.00062)
Heritability (%)	87.6	97.9	98.2
Descriptive statistics			
Mean	115.1547	68.43792	43.07698
Range	4.70 - 226.85	1.61 - 225.09	0.01 - 174.57
CV (%)	25.98	79.60972	108.0371
Correlation with TGSL			
Full panel	-	0.51 ***	0.08
Admixture Cluster 1	-	0.99 ***	-0.21
Admixture Cluster 2	-	0.22	0.56 ***

*** $p < 0.001$
