# Supplementary figures and images for: Genome-Wide Association Reveals Trait Loci for Seed Glucosinolate Accumulation in Indian Mustard (Brassica juncea L.)
Source: Plants (Basel). 2022 Jan 28;11(3):364. doi: 10.3390/plants11030364 (PMC8838242; doi:10.3390/plants11030364)

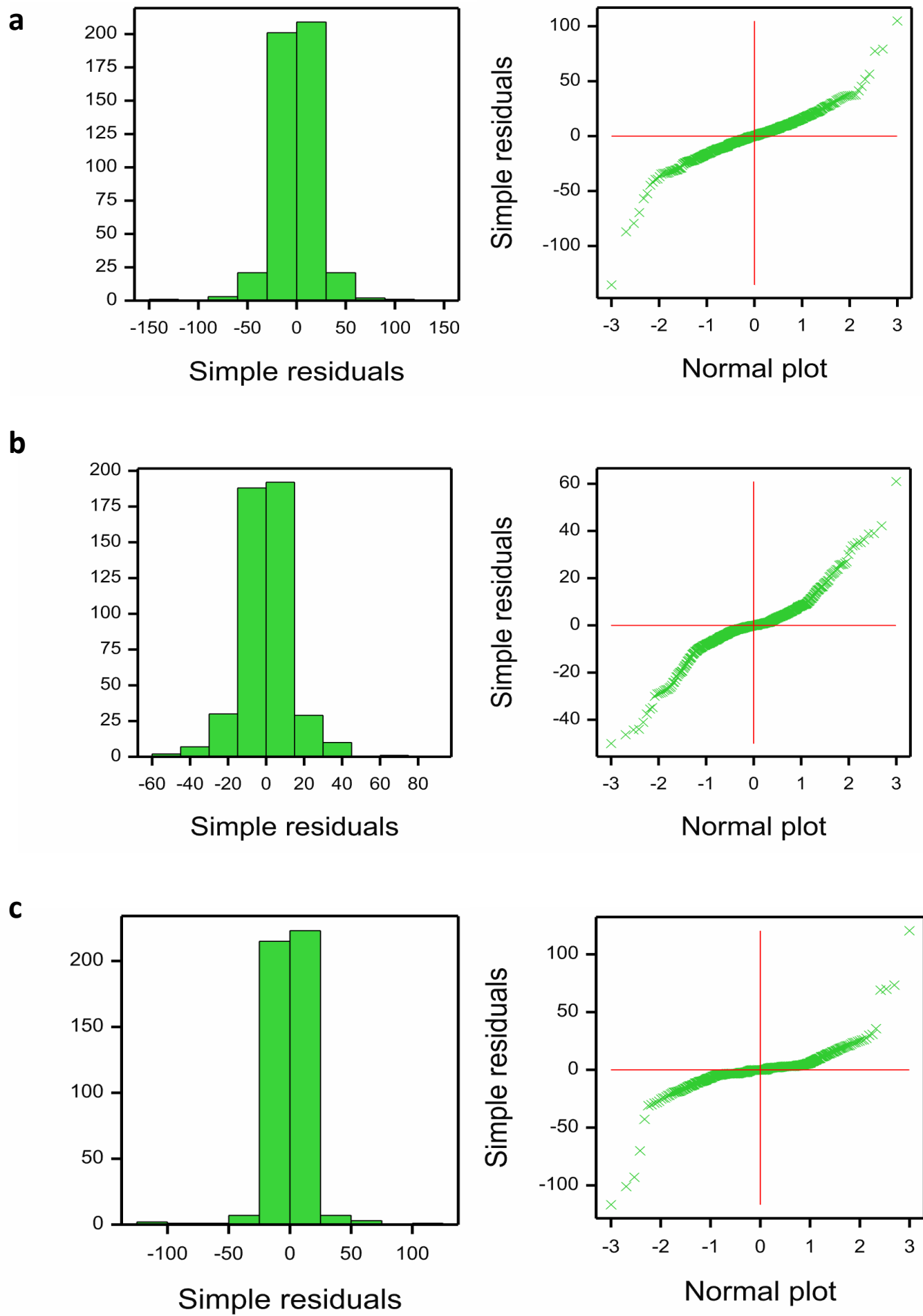

**Figure S1.** Residual distribution and normal plot for: **a)** total GSLs; **b)** sinigrin and; **c)** gluconapin.

Supplement: Supplementary file 1 [file plants-11-00364-s001.zip › Figure S1.pdf]
